# The efficacy of recombinant human soluble thrombomodulin for obstetric disseminated intravascular coagulation: a retrospective study

**DOI:** 10.1186/s13054-015-1086-3

**Published:** 2015-10-20

**Authors:** Masato Yoshihara, Kaname Uno, Sho Tano, Michinori Mayama, Mayu Ukai, Shinya Kondo, Tetsuya Kokabu, Yasuyuki Kishigami, Hidenori Oguchi

**Affiliations:** Department of Obstetrics, Perinatal Medical Center, TOYOTA Memorial Hospital, 1-1, Heiwa-cho, Toyota, Aichi Japan

## Abstract

**Introduction:**

Recombinant human soluble thrombomodulin (rhTM) is a novel anti-coagulant agent that regulates the imbalanced coagulation system by reducing the excessive activation of thrombin. rhTM potentially reduces the morbidity and mortality in patients with sepsis-induced disseminated intravascular coagulation (DIC). However, the efficacy of rhTM in obstetric DIC has not yet been established. We performed this study to examine whether the administration of rhTM was a potentially effective treatment for DIC induced by one or more underlying obstetric disorders.

**Methods:**

This is a single-center, retrospective cohort study conducted between January 2007 and February 2015 using the records of the Department of Obstetrics at the Perinatal Medical Center of TOYOTA Memorial Hospital, Aichi, Japan. The eligibility criteria were known or suspected obstetric DIC documented on the basis of clinical and laboratory data and association with one or more major underlying obstetric disorders. Baseline imbalance between patients with and without treatment of rhTM was adjusted using an inverse probability of treatment weighting using propensity scores composed of the following independent variables: severe postpartum hemorrhage, placental abruption, and preeclampsia/eclampsia, including hemolysis, elevated liver enzymes, and low platelet syndrome, initial platelet counts, D-dimer levels, fibrinogen levels, and prothrombin time–international normalized ratio (PT–INR). We evaluated laboratory changes and clinical outcomes in the early phase of obstetric DIC.

**Results:**

In total, 66 of 4627 patients admitted to our department during the study period fulfilled the required criteria; of these, 37 and 29 patients were included in the rhTM and control group, respectively. After adjustment, treatment with rhTM was associated with significant improvements in platelet counts, D-dimer levels, fibrinogen levels, and PT–INR compared with the control group. The platelet concentrate transfusion volume was significantly lower in the rhTM treatment group (3.02 vs 6.03 units, *P* = 0.016). None of the adjusted group differences were statistically significant for all types of organ damage and failure.

**Conclusion:**

rhTM administration was associated with clinical and laboratory improvement in patients with DIC caused by underlying obstetric conditions. Further clinical research is needed to clarify the optimal application of rhTM in each of the causative obstetric disorders.

## Introduction

Obstetric disorders are a major cause of disseminated intravascular coagulation (DIC), which increases maternal and fetal morbidity and mortality [1, 2]. In a study based on the United States Nationwide Inpatient Sample between 1998 and 2009, approximately one quarter of maternal deaths were related to obstetric DIC [3]. The most common obstetric complications associated with DIC are severe postpartum hemorrhage, placental abruption, and preeclampsia/eclampsia, including the syndrome of hemolysis, elevated liver enzymes, and low platelets (HELLP) [2]. In the management of DIC, the goal is to remove the underlying condition and provide intensive supportive care [1]. Although some drugs, such as heparin, antithrombin concentrates, or gabexate mesilate, control coagulation in the treatment of DIC, their efficacy remains uncertain [1, 4].

Recombinant human soluble thrombomodulin (rhTM) is a novel anti-coagulant agent composed of the active, extracellular domain of thrombomodulin (TM) that regulates the imbalanced coagulation system by reducing excessive activation of thrombin [5]. Additionally, rhTM has antifibrinolytic and anti-inflammatory properties, which are supposed to mitigate some of the catastrophic conditions of DIC [6]. In Japan, it has been reported that rhTM potentially reduces the morbidity and mortality in patients with sepsis-induced DIC [7–9]. Similarly, an international, randomized, placebo-controlled, phase IIb clinical trial of rhTM in patients with sepsis and suspected DIC [10] suggested that this treatment is both efficacious and safe, stimulating enthusiasm for the application of rhTM in critical care. On the other hand, the efficacy of rhTM in obstetric DIC has not yet been established. One recent study demonstrated the possible effectiveness of rhTM in postpartum hemorrhage with DIC [11]. However, obstetric DIC does not always comprise a single underlying disorder but often arises from multiple obstetric complications, some of which overlap and interact with each other. We performed this study to examine whether administration of rhTM is a potentially effective treatment for DIC induced by one or more underlying obstetric disorders.

## Materials and methods

### Study participants

We conducted a single-center, retrospective cohort study between January 2007 and February 2015 using the records of the Department of Obstetrics at the Perinatal Medical Center of TOYOTA Memorial Hospital, Aichi, Japan. This study was approved by the ethical committee of TOYOTA Memorial Hospital and was conducted in accordance with the principles of the Declaration of Helsinki. Because rhTM was approved for DIC, including obstetric DIC and available for market use in Japan, the committee did not require informed consent for this retrospective study.

Patients who met the following criteria were eligible: known or suspected obstetric DIC documented on the basis of clinical and laboratory data and association with one or more major underlying obstetric disorders, such as postpartum hemorrhage (total blood loss over 1000 mL), placental abruption, and preeclampsia/eclampsia, including HELLP syndrome. DIC was diagnosed using the DIC score of the Japanese Association for Acute Medicine (JAAM) or the obstetric DIC score approved by the Japanese Society of Obstetrics and Gynecology (Table 1) [12, 13]. In all patients the criteria for diagnosis of DIC were fulfilled by one or both of these scores.

**Table 1:** Obstetric DIC score

	Score
1. Underlying diseases	
a. Placental abruption	
Stiffening of the uterus, death of the fetus	5
Stiffening of the uterus, survival of the fetus	4
Confirmatory diagnosis of placental abruption by ultrasonic tomographic findings and CTG findings	4
b. Amniotic fluid embolism	
Acute cor pulmonale	4
Artificial ventilation	3
Assisted respiration	2
Oxygen flux alone	1
c. DIC-type postpartum hemorrhage	
In case the blood from the uterus has low coagulability	4
Hemorrhage of >2000 mL (within 24 hours after the start of hemorrhage)	3
Hemorrhage of 1000–2000 mL (within 24 hours after the start of hemorrhage)	1
d. Eclampsia	
Eclamptic attack	4
e. Severe infection	
Those with fever accompanied by shock, bacteremia, and endotoxemia	4
Continued fever or remittent fever	1
f. Other underlying diseases	1
2. Clinical symptoms	
a. Acute renal failure	
Anuria (<5 mL/hour)	4
Oliguria (5–20 mL/hour)	3
b. Acute respiratory failure (amniotic fluid embolism excluded)	
Artificial ventilation or occational assisted respiration	4
Oxygen flux alone	I
c. Organ failure	
Heart (rales or foamy sputum, etc.)	4
Liver (visible jaundice, etc.)	4
Brain (clouding of consciousness, convulsion, etc.)	4
Digestive tract (necrotic enteritis, etc.)	4
Other severe organ failure	4
d. Hemorrhage diathesis	
Macroscopic hematuria and melena, purpura, etc.	4
e. Shock symptoms	
Pulse rate ≥100/minute	1
Blood pressure ≤90 mm Hg (systolic) or reduction ≥40 %	1
Cold sweat	1
Pallor	1
3. Laboratory findings	
Serum FDP ≥10 pg/mL	1
Platelet counts ≤100 × 10^9^/L	1
Fibrinogen ≤150 mg/dL	1
PT ≥15 seconds (≤50 %) or hepaplastin test ≤ 50 %	1
Erythrocyte sedimentation rate of ≤4 mm/15 minutes or ≤15 mm/hour	1
Bleeding time ≥5 minutes	1
Other coagulation and fibrinolysis factors;	
antithrombin ≤18 mg/dL or ≤60 %, plasminogen, prekallikrein, other factors of ≤50 %	1
Diagnosis	
8–12 points	Suspected DIC
≥13 points	Definite DIC

*DIC* disseminated intravascular coagulation, *CTG* cardiotocography, *FDP* fibrin/fibrinogen degradation products, *PT* prothrombin time

The exclusion criteria were as follows: the presence of acute or chronic hepatic failure related to non-obstetric causes; known congenital coagulopathy diagnosed before pregnancy; underlying comorbidity, such as chronic renal failure, untreated diabetes mellitus, and malignancy, which could affect patient outcome; and missing clinical or laboratory data.

### Treatment

The management of patients with obstetric DIC focused on identifying and treating the underlying disorders and providing supportive care, which included mechanical ventilation, vasoactive agents, and blood products. All the patients underwent vaginal or cesarean delivery on the day in which DIC was diagnosed as being induced by an underlying obstetric disorder. In the patients with postpartum hemorrhage, DIC was assessed after delivery, while those with either placental abruption or preeclampsia/eclampsia were diagnosed before delivery.

In the rhTM group, rhTM was administered at a dose of 0.06 mg/kg/day via intermittent bolus over 30 minutes at the discretion of the attending physician after assessing the patient’s condition. The rhTM treatment was only initiated after delivery because rhTM was contraindicated for pregnant women, and it was discontinued after the improvement of both clinical conditions and laboratory parameters.

Blood transfusions were performed principally according to the management protocols for obstetric hemorrhage [14]. One unit each of red blood cells (RBCs), fresh frozen plasma (FFP), and platelet concentrate (PC) was equivalent to approximately 200 mL of whole blood. Fibrinogen concentrates were also used for the patients who required rapid replacement of coagulation factors. In some patients, protease inhibitors, such as antithrombin concentrates, gabexate mesilate, and nafamostat mesilate, were administered empirically. Some patients admitted to the ICU were treated with vasoactive agents such as norepinephrine and vasopressin or mechanical ventilation, as necessary.

### Data collection

We collected data on baseline characteristics, including the present obstetric status, associated underlying diagnosis, total postpartum hemorrhage volume, initial laboratory results, initial JAAM and obstetric DIC scores, and therapeutic interventions provided. The following outcome measures were recorded on the day in which the patient was diagnosed with obstetric DIC and approximately two days after diagnosis: platelet counts, D-dimer levels, fibrinogen levels, and prothrombin time–international normalized ratio (PT–INR). Within 4 days after the diagnosis of obstetric DIC, we also compiled data for the occurrences of bleeding events and DIC-related organ damage and failure (heart, lung, kidney, and liver) as well as for the number of units transfused of RBC, FFP, PC, and fibrinogen concentrates. We defined organ damage and failure as follows; heart, decreased ejection fraction (<50 %), or increased E/E’ (>8.0) with elevated serum brain natriuretic peptide level (>200 pg/mL); respiratory, fulfilling the criteria of acute respiratory distress syndrome, or requiring mechanical ventilation; renal, fulfilling the criteria of acute kidney injury; liver, increased serum liver enzyme levels (aspartate aminotransferase or alanine aminotransferase greater than three times the upper limit of normal).

### Statistical analysis

To assess the effect of the novel anticoagulant in this non-randomized experiment, we used a propensity score (PS) method in which the scores were estimated by fitting a multivariate logistic regression model to the original population of treated and untreated patients [15]. The independent variables that seemed to be strongly associated with the administration of rhTM were considered clinically and statistically relevant. Because of the specific characteristics of obstetric DIC and the small sample size of the retrospective cohorts, we included the following independent variables: severe postpartum hemorrhage, placental abruption, and preeclampsia/eclampsia, including HELLP syndrome, initial platelet counts, D-dimer levels, fibrinogen levels, and PT–INR. We adjusted the cohorts for the probability of treatment with the inverse probability weighting of treatment (IPTW) approach. Each individual was weighted by the inverse probability of receiving the treatment, equal to 1/PS for treated individuals or 1/(1− PS) for control individuals. With the IPTW method, no loss of sample occurs as that associated with alternative methods such as PS matching; therefore, this was considered beneficial for this small observational study [16, 17]. The standardized differences [18] of the independent variables before and after adjustment were calculated to evaluate the balances of these variables and the effectiveness of the PS-based IPTW analysis.

Comparisons between the groups were analyzed by Student’s *t* test or the Mann–Whitney *U* test for continuous variables as appropriate and by the chi-square or Fisher’s exact test for categorical variables. The changes in laboratory parameters from the baseline levels in the adjusted cohorts were analyzed by repeated measures analysis of variance adjusted for the baseline data as a covariate and by the post hoc Bonferroni test. The degrees of freedom were corrected using Greenhouse–Geisser estimates of sphericity. Homogeneity of variances was also examined with Levene’s test and the all parameters met the assumption. The differences in total transfusion volumes (RBC, FFP, PC, and fibrinogen concentrates) between the groups were assessed by the Kruskal–Wallis test before and after adjustment with the PS-based IPTW analysis. The effectiveness of rhTM for secondary organ damage and failure was also described as non-adjusted, and the IPTW-adjusted odds ratios (ORs) were estimated by logistic regression. Statistical significance was determined two-sided with *P* <0.05. All statistical analyses were carried out using IBM SPSS Statistics for Windows, Version 22.0 (IBM Corp., Armonk, NY, USA).

## Results

### Baseline characteristics of patients

In total, 66 out of 4627 patients admitted to our department during the study period fulfilled the required criteria; of these, 37 and 29 patients were included in the rhTM group and the control group, respectively. The baseline characteristics of the two groups were comparable (Table 2). There were no differences in obstetric history, delivery method, or underlying obstetric disorders between the two groups. The total amount of hemorrhage, initial laboratory results, JAAM DIC scores, and obstetric DIC scores were also comparable; however, the hemoglobin level differed (*P* = 0.013). For therapeutic intervention, the rates of ICU admission and protease inhibitor administration were not different between the two groups.

**Table 2:** Baseline characteristics of the patients

	rhTM group	Control group	*P* value^a^
	(n = 37)	(n = 29)	
Age, years, mean (SD)	33.0 (4.8)	31.8 (5.1)	0.326
Nulliparous, n (%)	20 (54.1)	16(55.1)	1.000
Twin pregnancy, n (%)	4 (10.8)	2 (6.9)	0.668
Delivery method, n (%)			
Normal vaginal delivery	10 (27.0)	7 (24.1)	1.000
Vacuum extraction/Forceps delivery	9 (24.3)	6 (20.7)	0.775
Cesarean section	18 (48.6)	16 (55.2)	0.628
Diagnosis, n (%)			
Severe postpartum hemorrhage^b^	31 (83.7)	19 (65.5)	0.147
Placental abruption^b^	16 (43.2)	12 (41.4)	1.000
Preeclampsia/eclampsia/HELLP syndrome^b^	7 (18.9)	10 (34.5)	0.169
Total hemorrhage volume, mL mean (SD)	2164.5 (1193.3)	1795.8 (1212.3)	0.221
Laboratory parameters, mean (SD)			
White blood count, × 10^3^/μL	16.75 (5.61)	16.63 (6.02)	0.937
Hemoglobin, g/dL	7.11 (2,00)	8.59 (2.70)	0.013
Platelet count, × 10^9^/L^b^	104.5 (51.9)	123.4 (71.8)	0.219
D-dimer, μg/mL^b^	116.3 (165.9)	116.6 (244.5)	0.995
Fibrinogen, mg/dL^b^	175.0 (94.4)	234.4 (138.4)	0.051
PT-INR^b^	1.346 (0.491)	1.426 (0.706)	0.587
JAAM DIC score, median (IR)	4.0 (3.0–6.0)	5.0 (2.0–7.0)	0.937
Obstetric DIC score, median (IR)	11.0 (9.0–14.0)	10.0 (8.0–13.0)	0.296
Theraputic intervention, n (%)			
ICU admission	10 (27.0)	8 (27.6)	1.000
Protease inhibitor	16 (43.2)	17 (58.6)	0.321

Data are expressed as group mean (SD), median (interquartile range (*IR*)), or proportion (%). ^a^ *P* value between the two groups from analysis using Student’s *t*–test or Mann–Whitney *U* test for continuous variables as appropriate, and by chi-square or Fisher’s exact test for categorical variables. ^b^The seven independent variables used in propensity score calculation. *rhTM* recombinant human soluble thrombomodulin, *HELLP* hemolysis, elevated liver enzymes, and low platelets, *PT–INR* prothrombin time–international normalized ratio, *JAAM* Japanese Association for Acute Medicine, *ICU* intensive care unit

### PS-based approach

PS was estimated for each individual by multivariate logistic regression modeling with the seven predetermined independent variables. The *P* value for the Hosmer–Lemeshow test of the PS model was 0.953, and that for the *c* statistic was 0.740. Using the IPTW approach, pseudo-populations were added for each group by weighting cases. Standardized differences, imbalances of independent variables between non-adjusted and adjusted cohorts, were reduced (Table 3). We also observed the balance of baseline characteristics of covariates within deciles of PS.

**Table 3:** Standardized differences of the independent variables

	Standardized difference	
	Non-adjusted	Adjusted
Severe postpartum hemorrhage	0.425	−0.020
Placental abruption	0.041	0.040
Preeclampsia/eclampsia/HELLP syndrome	−0.345	0.040
Platelet count	−0.302	−0.091
D-dimer	−0.001	0.086
Fibrinogen	−0.480	−0.090
PT-INR	−0.132	0.097

*HELLP* hemolysis, elevated liver enzymes, and low platelets, *PTxINR* prothrombin time–international normalized ratio

### Laboratory parameters

**Fig. 1:**
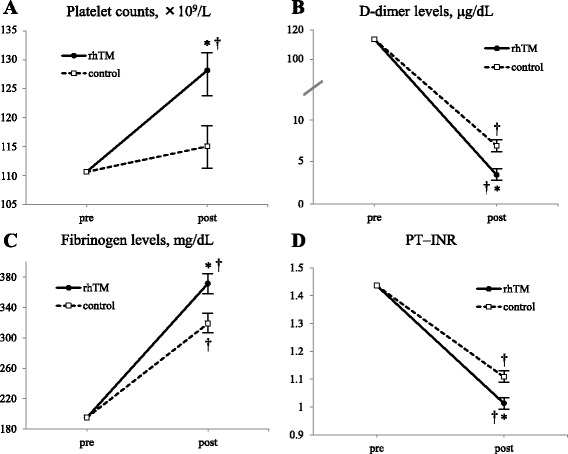
Changes in key laboratory parameters from baseline in platelet counts (**a**), D-dimer levels (**b**), fibrinogen levels (**c**), and PT–INR (**d**). Data are described as group mean ± standard error of the mean. The effect of treatment over time was also statistically significant in all the four parameters: *P <0.05 compared with the control group; ^†^ *P* <0.05 compared with baseline. *rhTM* recombinant human soluble thrombomodulin, *PT–INR* prothrombin time–international normalized ratio

Compared with the control group, platelet counts in the rhTM group were significantly increased from the baseline value at the post-treatment state approximately 2 days after the diagnosis (Fig. 1a). In terms of the coagulation parameters, D-dimer levels, fibrinogen levels, and PT–INR were significantly improved after the initiation of rhTM when compared with the control group (Fig. 1b, c, d). The effect of the treatment over time (from baseline to day 2) was also statistically significant for all four parameters.

### Blood transfusion

The total volumes of blood transfused within 4 days of diagnosis with DIC are presented in Table 4. In the original cohorts, there were no significant volume differences of the components of blood transfused between the two groups. However, when compared with the adjusted control group, the PC transfusion volume was significantly lower in the adjusted rhTM group (3.02 vs 6.03 units, *P* = 0.016). The other blood components were transfused at similar volumes among patients in the adjusted groups.

**Table 4:** Total transfusion amounts received by patients

	rhTM group	Control group	*P* value^a^
	(n = 37)	(n = 28)	
RBC, units, mean (median)			
Non-adjusted	5.65 (4.00)	4.83 (2.00)	0.164
Adjusted	4.90 (4.00)	5.62 (4.00)	0.876
FFP, units, mean (median)			
Non-adjusted	7.73 (5.00)	5.93 (5.00)	0.360
Adjusted	7.02 (5.00)	6.65 (5.00)	0.537
PC, units, mean (median)			
Non-adjusted	3.11 (0.00)	6.21 (0.00)	0.098
Adjusted	3.02 (0.00)	6.03 (0.00)	0.016
Fibrinogen, g, mean (median)			
Non-adjusted	0.32 (0.00)	0.17 (0.00)	0.822
Adjusted	0.49 (0.00)	0.22 (0.00)	0.706

Data are expressed as group mean (median). ^a^ *P* value between the two groups in the Kruskal–Wallis test. *RBC* red blood cells, *FFP* fresh frozen plasma, *PC* platelet concentrate

### Organ damage and failure related to DIC

DIC-related acute organ damage and failure was observed in some patients within 4 days after the onset of obstetric DIC (Table 5). In the original cohorts, liver damage was significantly less frequent in the rhTM group than in the control group (OR, 0.180; 95 % confidence interval, 0.034–0.944; *P* = 0.043). After adjustment, none of the group differences were statistically significant for any type of organ damage or failure.

**Table 5:** Comparison of organ damage and failure related to DIC among the original and adjusted cohorts

	rhTM group	Control group	Original cohorts		Adjusted cohorts	
	(n = 37)	(n = 29)	Odds ratio (95 % CI)^a^	*P* value^a^	Odds ratio (95 % CI)^a^	*P* value^a^
Cardiac, n (%)	1 (2.7)	1 (3.4)	0.778 (0.047–12.990)	0.861	0.970 (0.114–8.283)	0.978
Respiratory, n (%)	3 (8.1)	2 (6.9)	1.191 (0.186–7.645)	0.854	0.409 (0.115–1.460)	0.186
Renal, n (%)	3 (8.1)	3 (10.3)	0.765 (0.143–4.102)	0.754	0.737 (0.219–2.474)	0.621
Liver, n (%)	2 (5.4)	7 (24.1)	0.180 (0.034–0.944)	0.043	0.587 (0.216–1.595)	0.296

^a^Odds ratio and *P* value estimated by logistic regression analysis. *DIC* disseminated intravascular coagulation, *rhTM* recombinant human soluble thrombomodulin

### Adverse events

Three patients presented with a puerperal hematoma after vaginal delivery and treatment for massive postpartum hemorrhage, of which two occurred after rhTM administration. No other bleeding-related adverse events or death occurred during the study period.

## Discussion

In the present study, rhTM was associated with significant improvements in platelet counts, D-dimer levels, fibrinogen levels, and PT–INR when compared with those associated with the control group. The decreased PC transfusion volume also indicates the potential efficacy of rhTM in obstetric DIC. Specifically, by decreasing the imbalances between the two groups, the PS-based IPTW analysis enabled us to clarify this therapeutic effect. To our knowledge, this is the first retrospective cohort study to provide evidence of the potential efficacy of rhTM in DIC caused by underlying obstetric disorders.

The anticoagulant efficacy of rhTM has been described in DIC caused by the uncontrolled and excessive production of thrombin, leading to widespread and systemic intravascular fibrin deposition [1]. In vitro studies have shown that TM directly or indirectly inhibits further thrombin generation and inactivates the cleavage of fibrinogen and subsequent microvascular thrombus formation [19, 20]. The competitive inhibition of rhTM, which decreases the interaction between thrombin and fibrinogen, can directly decrease fibrinogen-to-fibrin degradation. Moreover, the relationship between thrombin and TM is reversible and much faster than that between thrombin and antithrombin, one of the most important natural anticoagulants [21]. This suggests that rhTM can exert its anticoagulant effects by reducing the speed of thrombin and antithrombin reaction, but without losing the core function of antithrombin. In addition, thrombin–rhTM complexes enhance protein C to generate activated protein C (APC), which serves as a negative regulator of the coagulation cascade through the inactivation of the cofactors Va and VIIIa [5]. In this study, we found significant improvements in platelet counts and coagulation parameters from the baseline values after treatment with rhTM, despite the same or lower requirement of blood transfusion. Given the evidence base, this suggests that rhTM suppressed the excessive consumption of platelets and coagulation factors. Therefore, each of the anticoagulant effects of rhTM appeared to act cohesively to improve the procoagulant state observed in DIC induced by obstetric underlying disorders.

TM binding to thrombin is also known to have antifibrinolytic efficacy by enhancing thrombin activatable fibrinolysis inhibitor (TAFI), the precursor of a basic carboxypeptidase (TAFIa), thereby stabilizing fibrin clots and ensuring localization to the sites of vascular injury. Fibrinolysis originates from the binding of plasminogen and tissue-plasminogen activator to the fibrin surface through the recognition of C-terminal lysine, the cleavage of which is induced by TAFIa [20, 22]. This can mediate the relatively profibrinolytic status arising from consumption coagulopathy, but without provoking hemorrhagic complications. During the peripartum period, bleeding sites are inevitably formed by injury to the parturient canal or by cesarean section. Besides, obstetric hemorrhage can be aggravated by bleeding diathesis in DIC [2]. In such a prohemorrhagic environment, rhTM does not appear to deteriorate the vicious profibrinolytic cycle induced by the activation of TAFI. However, this assumes that plasma replacement therapy is appropriately performed for the associated consumption coagulopathy, as demonstrated in our study.

In addition to its anticoagulant and antifibrinolytic effects, TM has anti-inflammatory properties, differentiating rhTM from other anticoagulants [19, 20]. TM binds to thrombin and mediates its proinflammatory activities to induce various chemical mediators. APC, enhanced by the TM–thrombin complex then independently inhibits the expression of several cytokines, resulting in inflammation being suppressed [19, 20, 23]. Under DIC conditions, excessive activation of coagulation yields proteases that influence systemic inflammation and produce extensive endothelial damage and multiple organ failure [1]. Therefore, in addition to the prompt elimination of the causes and of the ongoing thrombin generation, the administration of rhTM in the early phase of DIC seems to be able to minimize systemic inflammation and reduce secondary organ damage. In our analysis of the adjusted cohorts, none of the group differences were statistically significant for any type of organ damage or failure. Further trials are needed to determine the efficacy of rhTM for each type of organ damage and organ failure.

In each obstetric underlying disorder, rhTM can demonstrate its therapeutic effects with the following properties: severe postpartum hemorrhage, one of the most common causes of obstetric DIC, is associated with endothelial dysfunction caused by hypoxemia and metabolic acidosis. This, in turn, induces excessive tissue factor production and ultimately, consumption coagulopathy [2]. Although plasma replacement therapy is indispensable for the deficiency of coagulation factors, rhTM can prevent the progression of DIC by providing anticoagulant, antifibrinolytic, and anti-inflammatory effects. Placental abruption is another significant cause of obstetric DIC that is associated with increased maternal and fetal morbidity and mortality [2]. Herein, thrombin also plays a key role in the pathogenesis of the disease in the following manner: decidual bleeding and hypoxia induce the release of tissue factor, which generates abundant thrombin [24, 25]; this then leads to the expression of inflammatory cytokines and excess coagulation that overwhelms hemostatic control mechanisms, resulting in DIC [26]. It is prudent to initiate rhTM immediately after delivery and the control of bleeding as in case of severe postpartum hemorrhage. Preeclampsia is a pregnancy-specific multisystem disorder that is also a major cause of DIC in obstetrics [27]. The pathophysiology of preeclampsia is still uncertain, but some observational studies have reported that placental underperfusion, hypoxia, and ischemia induce circulating antiangiogenic factors that create widespread maternal endothelial dysfunction, resulting in hypertension and proteinuria [28]. The progression of endothelial dysfunction can also trigger DIC via the release of proinflammatory cytokines and via the activation of extrinsic coagulation pathways [2]. Eclampsia and the HELLP syndrome are considered the most severe subtypes of preeclampsia, which can cause critical brain and liver dysfunction and other severe maternal and fetal sequelae [29]. In combination with the termination of pregnancy, rhTM can biochemically mediate the associated endothelial dysfunction through its anticoagulant and anti-inflammatory properties.

Although there might also be some concern about the increased risk of bleeding, the critical concentration at which bleeding occurs is much higher than the recommended dose, and there has been no clinical evidence of a definite, direct interaction between rhTM administration and increased risk of bleeding-related complications in obstetric DIC [30]. In this study, a puerperal hematoma was a reported bleeding-related adverse event in three patients (two patients in the rhTM group and one patient in the control group), but it is uncertain whether this was because of the administration of rhTM or DIC itself. Additionally, it is important to remember that treatment with rhTM should be discontinued after confirming the improvement of clinical and laboratory variables such as vital signs, platelet counts, and coagulation parameters.

The limitations of this study include the lack of universal applicability of the obstetric DIC score and the existence of confounding factors between the groups because of the retrospective study design. Although we introduced a PS-based analysis to reduce the imbalance between the original cohorts in the probability of the rhTM treatment, some unknown confounding factors may have been missed. Due to the difficulty of extrapolating this retrospective analysis directly to any recommendations for treatment, the results of this pilot study should be the basis for additional studies, including randomized trials to examine the efficacy and safety of rhTM for obstetric DIC.

## Conclusions

In conclusion, rhTM administration was associated with clinical and laboratory improvement in patients with DIC caused by underlying obstetric conditions. Further clinical research is needed to clarify the optimal application of rhTM in each of the causative obstetric disorders, preferably in a multicenter, prospective cohort study, or randomized trials with sufficient power to confirm the results of this study.

## Key messages

- To our knowledge, this is the first retrospective cohort study to provide evidence of the efficacy of rhTM in DIC caused by underlying obstetric disorders
- When compared with the control group, treatment with rhTM was associated with significant improvements in platelet counts, D-dimer levels, fibrinogen levels, and PT–INR
- The decreased PC transfusion volume also indicates the potential efficacy of rhTM in obstetric DIC
- None of the adjusted group differences were statistically significant for any type of organ damage or organ failure
- By decreasing the imbalances between the two groups, the PS-based IPTW analysis enabled us to clarify the therapeutic effects. However, a multicenter prospective cohort study is needed to clarify the optimal application of rhTM in each obstetric disorder and to confirm the results of this study

## Abbreviations

DIC: disseminated intravascular coagulation
FFP: fresh frozen plasma
HELLP: hemolysis, elevated liver enzymes, and low platelets
ICU: intensive care unit
IPTW: inverse probability weighting of treatment
JAAM: Japanese Association for Acute Medicine
OR: odds ratio
PC: platelet concentrate
PS: propensity scores
PT–INR: prothrombin time–international normalized ratio
RBC: red blood cells
rhTM: recombinant human soluble thrombomodulin
TAFI: thrombin activatable fibrinolysis inhibitor
TAFIa: activated thrombin activatable fibrinolysis inhibitor
TM: thrombomodulin
